# Effects of a Virtual Reality Game With Leap Motion Controller on Brain Activity Related to Attentional Function in Healthy Adults - A Pilot EEG Study

**DOI:** 10.7759/cureus.71838

**Published:** 2024-10-19

**Authors:** Hiroki Annaka, Tamon Hiraoka, Tomonori Nomura

**Affiliations:** 1 Department of Occupational Therapy, Niigata University of Health and Welfare, Niigata, JPN; 2 Graduate School, Niigata University of Health and Welfare, Niigata, JPN; 3 Department of Occupational Therapy, Faculty of Rehabilitation, Niigata University of Health and Welfare, Niigata, JPN

**Keywords:** attentional function, cognitive function, cognitive rehabilitation, leap motion controller, virtual reality game

## Abstract

Objective

Virtual reality (VR) games with the Leap Motion Controller (LMC) are used in clinical practice for cognitive rehabilitation to improve attentional function. However, the effects of VR games using the LMC on brain activity related to attentional function have not yet been elucidated. This study aimed to elucidate the effects of a VR game with the LMC on brain activity related to attentional function.

Methods

This single-arm study included 15 healthy adults (mean age, 23.2 ± 0.9 years; seven females) and analyzed their electroencephalography (EEG) findings. We measured event-related potentials (ERPs) before and after the VR game with the LMC, and the α, θ, and β values during the VR game with the LMC. EEG measurements were based on the international 10-20 system, with dish electrodes placed at F3, F4, Fz, C3, C4, Cz, P3, P4, Pz, O1, and O2 using EEG caps. For statistical analysis, the N1 peak amplitudes before and after the VR game were compared using paired t-tests. In addition, the average amplitudes of α, θ, and β at 10-20 ms and 280-290 ms during the VR game were compared using paired t-tests only for positions that showed significant changes in the N1 peak amplitude before and after the VR game.

Results

After the VR game, the N1 peak amplitude was significantly greater at the F4 (pre: -4.51 ± 3.49 μV, post: -6.31 ± 2.60 μV, P = 0.010), Fz (pre: -4.68 ± 2.85 μV, post: -6.29 ± 2.17 μV, P = 0.033), and C4 (pre: -4.43 ± 3.94 μV, post: -6.24 ± 2.56 μV, P = 0.015) positions. During the VR game, the average amplitude of α at the F4 position (during a VR game of 10-20 ms: 7.86 ± 1.33 μV; during a VR game of 280-290 ms: 9.40 ± 2.69 μV, P = 0.010) was significantly higher at 280-290 ms.

Conclusions

The N1 peak amplitude, meaning selective attention, in F4, Fz, and C4 and the amplitude of α, meaning control interference of unwanted stimuli in a task, in F4 were increased in the post-VR game using LMC. VR games using the LMC may contribute to improvements in attentional function. Further studies are required to assess their applications in cognitive rehabilitation.

## Introduction

Virtual reality (VR) games are rapidly gaining popularity as a new rehabilitation method [1,2]. Among these games, VR games that use the Leap Motion Controller (LMC) have attracted attention because they are inexpensive, simple, and allow patients to be independent. The LMC uses infrared sensors to project a player's hands and fingers onto a monitor and reproduce hand and finger movements on the screen. Owing to this characteristic, VR games using the LMC are used for rehabilitation of upper limb function [3,4]. Interestingly, recent studies have reported that VR games using the LMC improve cognitive and attentional functions [5]. However, the mechanism by which VR games using the LMC improve cognitive function has not yet been elucidated or applied to cognitive rehabilitation. Attentional function is the foundation of cognitive function and essential for independence in daily life, and the use of cognitive rehabilitation to improve attentional function is still under investigation [6,7]. The application of VR games using the LMC, which are inexpensive and simple, in cognitive rehabilitation may improve the cognitive function of many patients.

For the application of VR games in rehabilitation, the effect on brain activity is an important consideration [8,9]. Head-mounted VR games have been used for cognitive rehabilitation by verifying their effects on brain activity based on event-related potentials (ERPs) and frequency bands determined with the international 10-20 system [8]. In particular, the ERP of the N1 peak amplitude and the amplitudes of α, θ, and β, which represent attentional function, are considered important for understanding changes in brain activity related to cognitive function [8,10]. However, very few studies have examined the effects of VR games using the LMC on brain activity, and the existing studies on this topic have examined changes in brain activity related to upper limb function [11,12]. To apply VR games using the LMC in cognitive rehabilitation for attentional function, their effects on brain activity related to attentional function have to be verified.

Therefore, this study aimed to investigate how a VR game using the LMC affects specific brain regions associated with selective attentional functions, using EEG analysis, and to explore its potential applications in cognitive rehabilitation. We examined the changes in the ERPs of N1 before and after the VR game as well as the changes in the amplitude of α, θ, and β during the VR game. 

## Materials and methods

Participants

Seventeen healthy adults participated in this study. Two of them were excluded due to missing electroencephalography (EEG) data, and the data for the remaining 15 healthy adults (mean age, 23.2 ± 0.9 years; seven females) were included in the analysis. The inclusion criteria were (a) right-handedness according to the Edinburgh Handedness Inventory [13] and (b) native Japanese. The exclusion criteria were (a) an intelligence quotient of ≤80 as measured by the Japanese Adult Reading Test [14], and (b) a diagnosis of upper limb dysfunction, neurological disease, psychiatric disease, developmental disability, color blindness, or visual field defects. All participants were instructed to refrain from caffeine and drug intake on the day of the experiment.

All participants provided written informed consent prior to participation in the study. The study was conducted in accordance with the Declaration of Helsinki, and the study protocol was approved by the Ethics Committee of Niigata University of Health and Welfare (approval no. 19262-240422). This study was registered at the University Hospital Medical Information Network Center (UMIN000053853).

Protocol

Measurements of ERPs before and after the VR game were performed in a soundproof room. The task was performed with the participant seated in a chair with a backrest and head support at a distance of 11.81 inches from the computer used to display the task. The participants performed the task while wearing earplugs.

**Figure 1:**
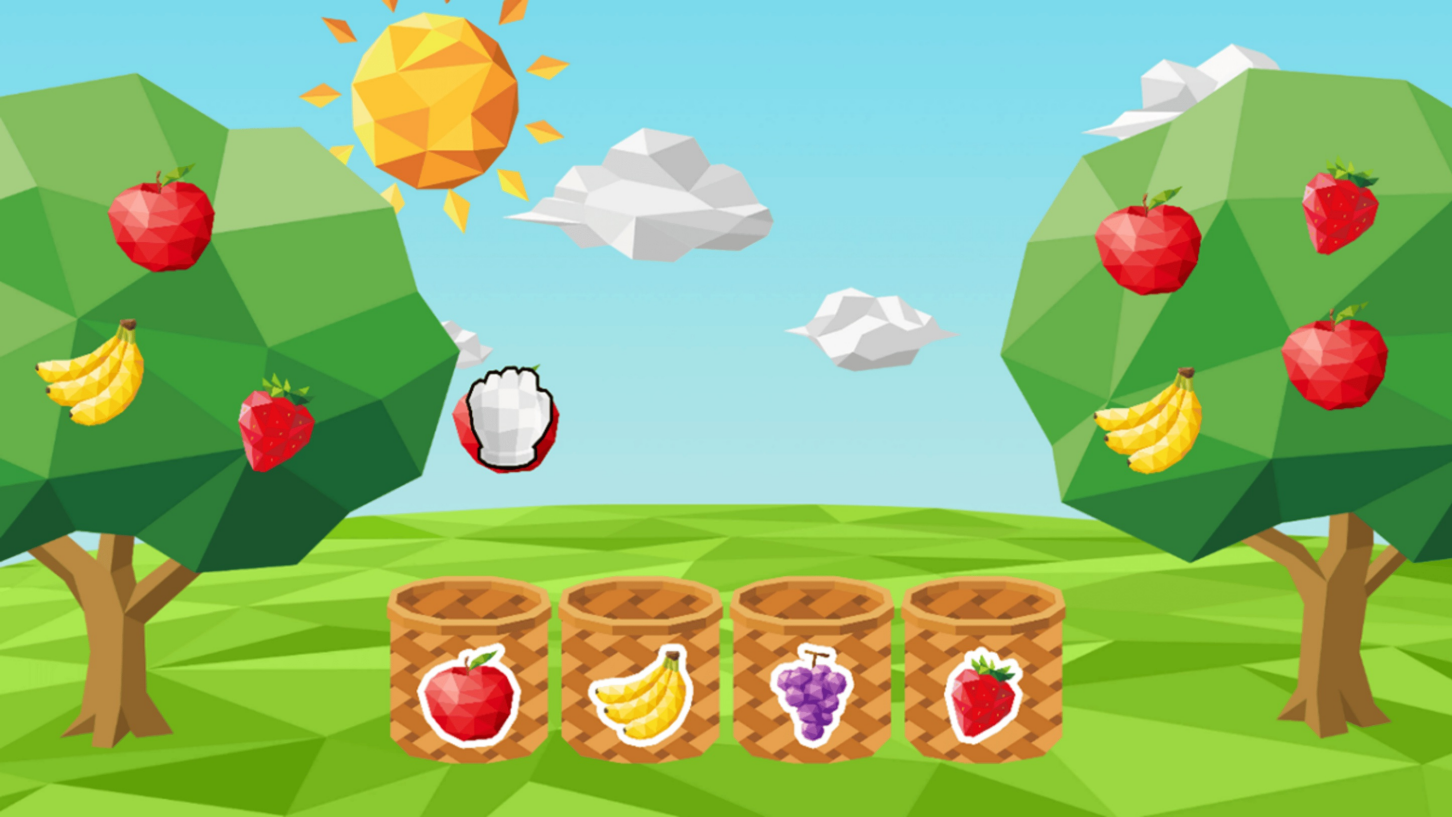
“Sunny Fruit Picking,” a virtual game using a Leap Motion Controller． The Leap Motion Controller controlled the hand projected onscreen to place the fruits in the baskets.

The VR game using the LMC was “Sunny Fruit picking” (Digireha, Tokyo, Japan) by Digital Interactive Rehabilitation System (Figure 1). In this game, the player manipulated a hand projected on the screen through the LMC to add as many fruits into the baskets as possible within 5 min. A player's hand is captured by holding it over the LMC on the table. The LMC was manipulated with the right hand. This VR game allows the player to adjust the sensitivity of the LMC to detect the player's hand movements, the size of the hand on the monitor, and the size of the target fruit at five different levels. All settings were set to level 3 in the study. The player is required to select from the multiple fruits on the screen, grab the fruit, and place it in the basket with the same fruit baskets. This task gives the player an immersive experience and requires an attentional function. Participants took a five-minute break before and after playing the VR game using the LMC.

**Figure 2:**
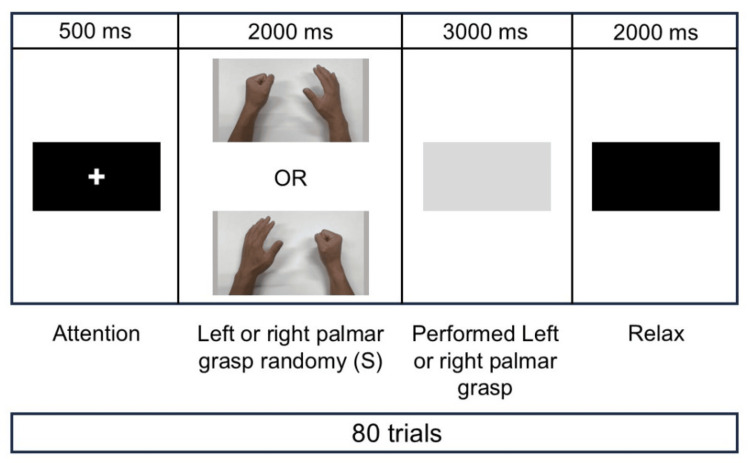
The event-related potential paradigm. S: stimulus.

The ERP paradigm was based on a previous study (Figure 2) [15]. For this assessment, a set of four images was presented across 80 trials. The first image was presented for 500 ms on a black background with a white “plus” in the center. When the image was presented, the participant’s attention was directed toward the screen. The second image was presented for 2000 ms with either the left or right hand clasped. This image was defined as the stimulus (S), and the participant identified the hand being held using the left or right hand. The third image was a gray image in which the participant held the hand on the same side as the hand held in the second image. The fourth image was a black screen. At this time, the participants were allowed to relax and blink.

EEG

EEG was performed using Polymate Pro MP6100 (Miyuki-Giken, Tokyo, Japan). The regions of interest were F3, F4, Fz, C3, C4, Cz, P3, P4, Pz, O1, and O2 based on the international 10-20 system, and the dish electrode was placed using an EEG cap. The sampling frequency and band-bus filter were set to 245 Hz and 0.5-30 Hz, respectively. For preprocessing to remove external interference, the notch filter was set to 50 Hz. The reference electrode was placed on both earlobes. Electrooculography (EOG) and surface electromyography (EMG) were performed for artifact removal.

Data processing Electro Magnetic Source Estimation (Cortech Solutions, Wilmington, NC) was used to analyze the ERPs. The analysis epoch ranged from 200 ms before the stimulus (S) presentation to 2000 ms after the stimulus (S) presentation (Figure 2). Averaging was performed after the removal of apparent artifacts from the EOG and surface EMG. N1 was defined as 90-150 ms after the stimulus (S) presentation and the peak amplitude of this epoch was recorded [16].

The amplitudes per second of α (4-7 Hz), θ (8-13 Hz), and β (14-30 Hz) during the VR game were calculated using EEG CDM analysis (Miyuki-Giken, Tokyo, Japan). Peak-to-peak values were set at 100 μV to exclude EOG and EMG artifacts. The average amplitudes of α, θ, and β were calculated at 10-20 ms and 280-290 ms during the VR game.

Statistical analysis

Statistical analysis was conducted using the IBM Statistical Package for the Social Sciences Statistics for Windows, Version 27.0 (IBM Corp, Armonk, NY). The Shapiro-Wilk test confirmed the normality of the N1 peak amplitude and the averaged amplitudes of α, θ, and β. The N1 peak amplitudes before and after the VR game were compared using a paired t-test. When the N1 peak amplitudes before and after the VR game showed significant changes, the averaged amplitudes of α, θ, and β at 10-20 and 280-290 ms during the VR game were compared using a paired t-test to understand the changes in brain activity during the VR game.

Sample size

The sample size was calculated using G*Power version 3.1.9.4 (Düsseldorf, Germany) with the following parameters: test family: t-test, statistical test: Difference from constant (one-sample case), tails: two, effect size: 0.8, α error: 0.05, and power: 0.80. The required sample size for this experiment was determined to be 15.

## Results

**Figure 3:**
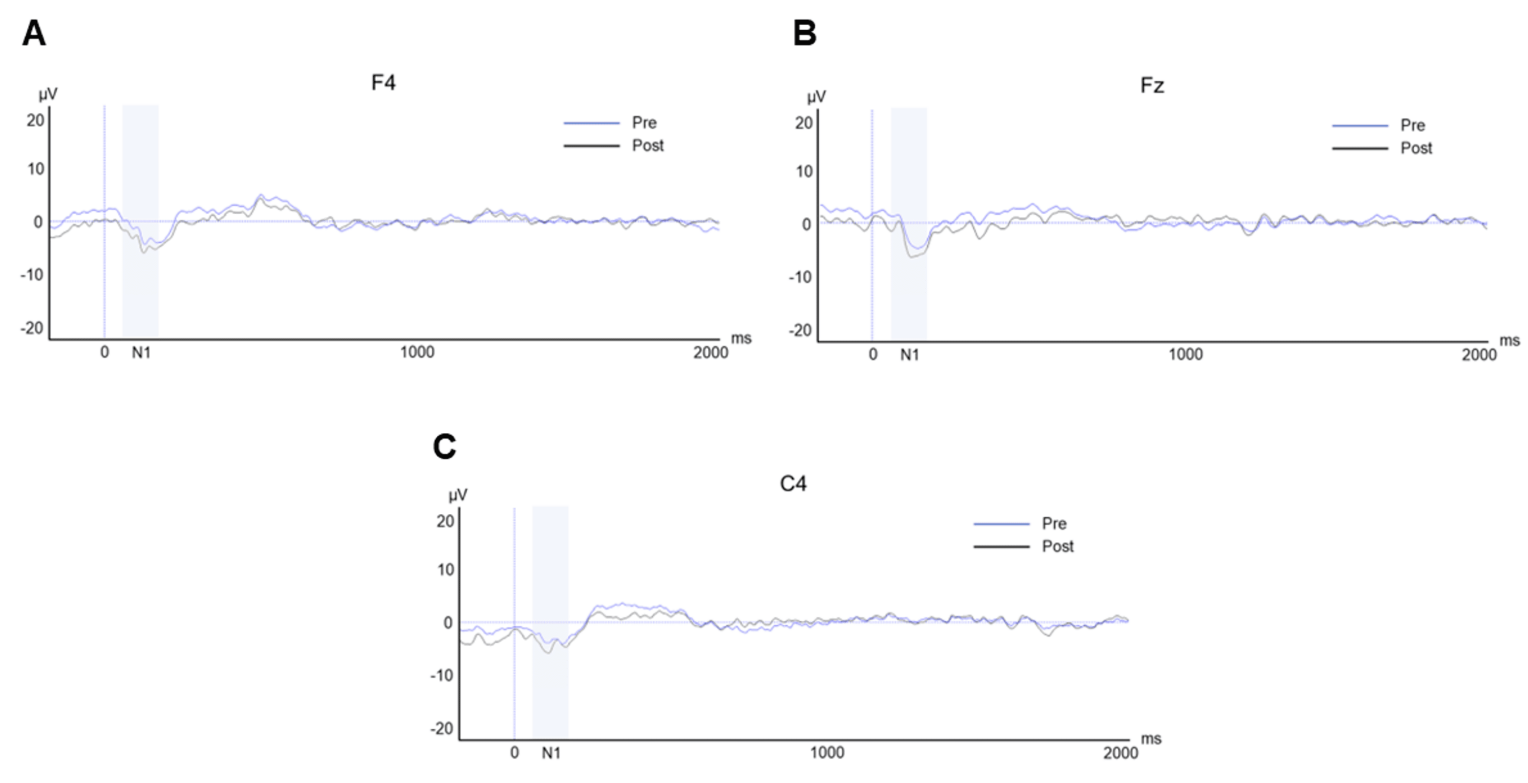
N1 peak amplitudes before and after the virtual reality game. Pre: before the virtual reality game; post: after the virtual reality game. Vertical axis: amplitude; horizontal axis: time. A: F4; B: Fz; C: C4. Epoch: -200 to 2000 ms. N1: 90-150 ms: fill area.

Table 1 shows the N1 peak amplitudes before and after the VR game. The N1 peak amplitudes at F4 (pre-game: -4.51 ± 3.49 μV, post-game: -6.31 ± 2.60 μV, P = 0.010), Fz (pre-game: -4.68 ± 2.85 μV, post-game: -6.29 ± 2.17 μV, P = 0.033), and C4 (pre-game: -4.43 ± 3.94 μV, post-game: -6.24 ± 2.56 μV, P = 0.015) increased significantly after the VR game (Figure 3).

**Table 1:** Changes in N1 peak amplitudes after the virtual reality game. P < 0.05. Position: position based on the international 10-20 system.

Position	Pre-game N1 peak amplitude (μV)	Post-game N1 peak amplitude (μV)	t-Value	P-value
F3	-4.71 ± 3.54	-6.01 ± 2.29	2.10	0.053
F4	-4.51 ± 3.49	-6.31 ± 2.60	2.99	0.010
Fz	-4.68 ± 2.85	-6.29 ± 2.17	2.07	0.033
C3	-5.50 ± 3.56	-5.08 ± 3.81	-0.42	0.675
C4	-4.43 ± 3.94	-6.24 ± 2.56	2.76	0.015
Cz	-6.65 ± 3.06	-7.10 ± 2.38	0.79	0.443
P3	-5.62 ± 4.63	-6.32 ± 3.45	1.09	0.294
P4	-6.50 ± 4.36	-6.47 ± 3.50	-0.05	0.959
Pz	-6.84 ± 4.32	-6.85 ± 3.73	0.02	0.984
O1	-2.89 ± 4.30	-3.17 ± 3.81	0.66	0.518
O2	-1.76 ± 4.25	-2.79 ± 3.82	2.07	0.057

**Figure 4:**
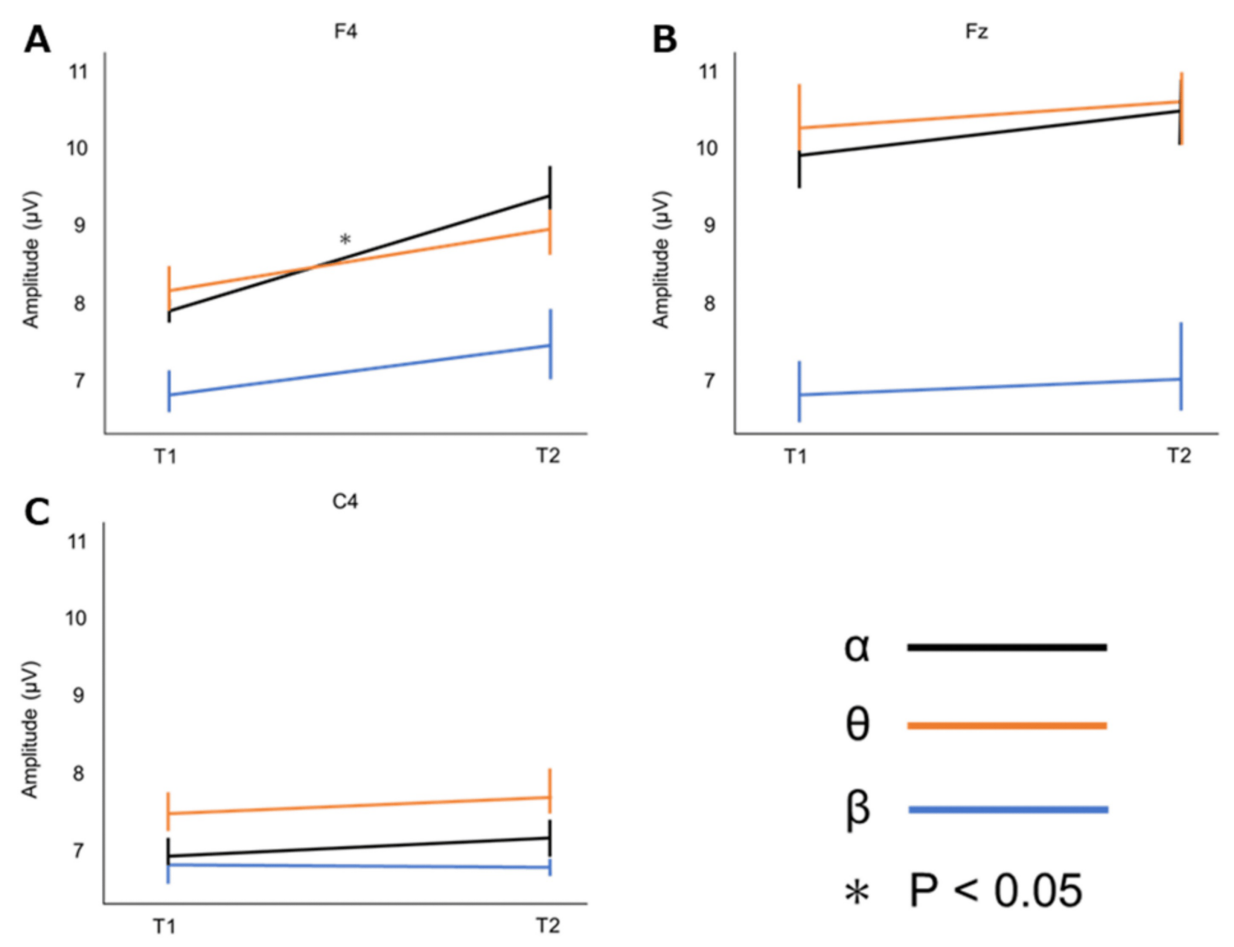
Changes in amplitude during the virtual reality game. T1: Average amplitude at 10-20 ms during the virtual reality game; T2: Average amplitude at 280-290 ms during the virtual reality game. A: F4; B: Fz; C: C4.

Table 2 shows the average amplitudes of α, θ, and β at 10-20 and 280-290 ms in F4, Fz, and Cz during the VR game. The average amplitude of α in F4 (at 10-20 ms during the VR game: 7.86 ± 1.33 μV, at 280-290 ms during the VR game: 9.40 ± 2.69 μV, P = 0.010) was significantly higher at 280-290 ms during the VR game (Figure 4).

**Table 2:** Changes in amplitude during virtual reality game. P < 0.05. Position: position based on the international 10-20 system. VR: virtual reality.

Position	Wave	Average amplitude at 10-20 ms during VR game (μV)	Average amplitude at 280-290 ms during VR game (μV)	t-Value	P-value
F4	α	7.86 ± 1.33	9.40 ± 2.69	-2.97	0.010
	θ	8.63 ± 1.80	9.74 ± 3.88	-1.52	0.151
	β	6.25 ± 1.18	7.13 ± 2.98	-1.42	0.177
Fz	α	9.44 ± 2.14	10.48 ± 3.32	-1.07	0.301
	θ	10.44 ± 2.95	10.69 ± 3.11	-2.54	0.803
	β	6.69 ± 1.56	6.70 ± 1.75	-0.26	0.980
C4	α	7.28 ± 1.51	7.93 ± 2.10	-1.97	0.068
	θ	7.34 ± 1.70	7.51 ± 1.67	-0.56	0.933
	β	6.48 ± 1.50	6.45 ± 1.75	0.08	0.057

None of the participants experienced any difficulty in operating the LMC during the VR game.

## Discussion

This pilot EEG study examined the effects of a VR game using the LMC on brain activity. The N1 peak amplitude increased at F4, Fz, and C4 after the VR game using the LMC. Furthermore, the average amplitudes of α at F4 increased during the VR game. These results suggest that VR games using the LMC may improve attentional function.

The present study showed an increase in the N1 peak amplitude at F4, Fz, and Cz after the VR game using the LMC. An increased N1 peak amplitude is recognized as an indicator of improved attentional function [10,17]. The N1 peak amplitude in the frontal region represents an early attentional process to the stimulus and is particularly related to selective attention to the stimulus. An increase in N1 peak amplitude indicates improved selective attention. The N1 peak amplitude in the central region represents self-inhibition of the activated response to stimuli [18]. The N1 of this position appears just before the behavior, and the more inhibited it is, the more the peak amplitude increases. Furthermore, the frontal and central networks have an attentional function that inhibits the activation of task-irrelevant behaviors [19]. The results of this study suggest that VR games using the LMC may have activated these attentional functions. A previous study reported that the N-back task after a head-mounted VR game showed increased N1 peak amplitudes in the frontal and central positions [20]. This is because immersive VR games enhance concentration on the stimuli [20]. VR games using the LMC are immersive because they require players to judge their behavior in response to stimuli and perform tasks using their own hand movements. These characteristics may have led to an increase in N1 peak amplitude.

To understand in detail the mechanism underlying the improvement in attentional function, we evaluated the changes in the amplitudes of α, θ, and β during the VR game in positions that showed changes in N1 peak amplitude. The analysis results showed that the α of F4 increased during the VR game using the LMC. Head-mounted VR games have been reported to increase the amplitude of α waves in the frontal cortex [21]; however, this has not been tested in VR games using the LMC. Task performance requires suppression of the cognition of irrelevant stimuli [22]. Alpha controls the interference of unwanted stimuli in a task and increases in amplitude when this function is activated [23]. Thus, the mechanism by which the N1 peak amplitude increased may involve the improvement of attentional function in the frontal lobe by VR games using the LMC. In contrast, the results of this study did not show an increase in the amplitude of θ and β. Previous studies have reported different roles for α, θ, and β in cognitive function [24]. θ and β are activated during higher cognitive performance, such as working memory [25]. The VR game used in this study is a simple task and does not require higher-order cognitive performance such as working memory. This feature of the game may be the reason why only the amplitude of α is increased. Differences in the effects of different types of VR games using the LMC on brain activity need to be investigated in the future.

To date, VR games using the LMC have not been fully explored as a cognitive rehabilitation of attentional function. The novelty of this study was to examine the potential application of VR games using the LMC for cognitive rehabilitation referencing EEG, which can objectively and quantitatively evaluate brain activity related to attentional function. Previous studies have reported that VR games are effective in improving attentional function [26,27]. However, many VR gaming devices may not be applicable to older adults and patients with stroke or developmental disabilities owing to their high cost, high game difficulty, and difficulty in equipment preparation. In contrast, a VR game using the LMC is inexpensive, simple, and requires only a USB cable connected to a PC for preparation. Thus, VR games using the LMC may have clinical applications in patients who have difficulty with cognitive rehabilitation using conventional VR games.

Limitation

The study has some limitations and the results should be treated with caution. Owing to the single-arm design, no comparisons with a non-VR game group using the LMC were performed. In addition, changes in attentional performance were not measured. Further research is required to examine the differences in performance changes in attentional function between VR game implementations with and without the LMC. Second, this study was conducted in healthy adults, and experiments on patients are required to validate the clinical application of this approach. Third, this study only measured the pre- and post-game N1 peak amplitudes; therefore, the duration of brain activation by the VR game using the LMC was not confirmed. Fourth, we did not examine whether the effect of VR games on brain activity changes with time or frequency. Further research will examine the persistence of changes in brain activity as the conditions under which this VR game is played vary. Fifth, we may not have adequately assessed the other brain regions for attentional function according to our electrode placement in the international 10-20 system. Further studies are needed using the international 10-10 system. Sixth, the effect of VR games using LMC on left-handed participants needs to be examined. Finally, the small sample size of this study may have prevented an adequate examination of the effects of individual variability in attentional function and sex. 

## Conclusions

This study examined the effects of a VR game with the LMC on brain activity by using EEG. The N1 peak amplitude at F4, Fz, and C4 and the amplitude of α at F4 increased after the VR game using the LMC. 

In comparison with other VR games, this VR game may be useful for cognitive rehabilitation in older adults, stroke patients, and those with developmental disabilities. However, this pilot study has several limitations for generalization. Further studies are required to examine the applications of this VR game in cognitive rehabilitation.
